# Immune Responses against the Omicron Variant of SARS-CoV-2 after a Third Dose of COVID-19 Vaccine in Patients Living with Human Immunodeficiency Virus (PLWH): Comparison with Healthcare Workers

**DOI:** 10.3390/vaccines10122129

**Published:** 2022-12-13

**Authors:** Joung Ha Park, Hyemin Chung, Min-Chul Kim, Seong-Ho Choi, Jin-Won Chung

**Affiliations:** 1 Division of Infectious Diseases, Department of Internal Medicine, Chung-Ang University Gwangmyeong Hospital, Gwangmyeong 14353, Republic of Korea; 2 Division of Infectious Diseases, Department of Internal Medicine, Chung-Ang University Hospital, Seoul 06973, Republic of Korea

**Keywords:** humoral immunity, cellular immunity, SARS-CoV-2, omicron, patient living with HIV (PLWH), health personnel

## Abstract

We compared immune responses against the omicron variant of SARS-CoV-2 after a third dose of the coronavirus disease 2019 (COVID-19) vaccine between people living with human immunodeficiency (PLWH) and healthcare workers (HCWs). In this prospective observational study, PLWH and HCWs vaccinated with at least two doses of vaccine were enrolled. We analyzed neutralizing responses using the GenScript SARS-CoV-2 surrogate virus neutralization test kit. Twenty-nine PLWH and 114 HCWs were included to analyze immune responses after the third vaccination. Most PLWH (86.2%) had fully suppressed viral loads and CD4 T cell counts were well-controlled (median 670.0 cells/μL). The neutralizing responses against the omicron variant in PLWH were not significantly different from those in HCWs (43.94% vs. 51.77%, *p* = 0.42). However, neutralizing responses against the omicron variant were significantly impaired by about 50% compared with wild type SARS-CoV-2 in PLWH (43.94% vs. 97.46%, *p* < 0.001) and HCWs (51.77% vs. 97.74%, *p* < 0.001). Although neutralizing responses against the omicron variant in well-controlled PLWH were comparable to those of HCWs, the responses were much lower than those against wild type in both PLWH and HCWs. Therefore, the risk of breakthrough SARS-CoV-2 infection due to the currently circulating omicron variant is still high despite three doses of vaccine in PLWH and will not differ from HCWs.

## 1. Introduction

People living with human immunodeficiency virus (PLWH) are at increased risk of severe coronavirus disease 2019 (COVID-19) because of their common co-morbidities and immune-compromised status [1,2]. Thus, vaccination is one of the key strategies to prevent COVID-19 in PLWH. It is known that vaccine-induced immune responses might be impaired and wane rapidly in PLWH compared with the general population as a result of chronic immune dysregulation and inflammation, even if PLWH have well-controlled status with antiretroviral therapy (ART) [3,4]. However, previous studies revealed that immune responses after two doses of the COVID-19 vaccine in PLWH were comparable to those of healthy controls if human immunodeficiency virus (HIV) viral loads were well suppressed and CD4 T cell counts were within the normal range by antiretroviral therapy [5,6,7]. Currently, based on these data, the Centers for Disease Control and Prevention recommends two doses of vaccine as a primary series in well-controlled PLWH, and three doses for those who have advanced HIV or are not taking antiretroviral therapy [4,8,9].

The omicron variant (B.1.1.529) of SARS-CoV-2 emerged in November 2021 and rapidly replaced the delta variant (B.1.617.2) worldwide. Because the omicron variant has more than 30 mutations in the spike protein of the virus, and at least 15 of these are in receptor binding domain (RBD), vaccine effectiveness against the omicron variant is considerably lower than for wild type SARS-CoV-2 and prior variants [10]. Previous studies demonstrated that a third dose of the mRNA COVID-19 vaccine elicited a significant increase in neutralizing responses against the omicron variant in the general population [11,12,13]. However, few studies have described humoral responses after a third dose of the COVID-19 vaccine in PLWH [14]. Thus, we evaluated humoral responses including neutralizing antibodies against the wild type and omicron variant of COVID-19, and compared the responses between PLWH and healthcare workers without HIV (HCWs) after three vaccinations. We also evaluated cellular responses by measuring interferon-gamma (IFN-γ) production against the omicron variant in PLWH.

## 2. Materials and Methods

### 2.1. Study Population and Study Design

**Figure 1:**
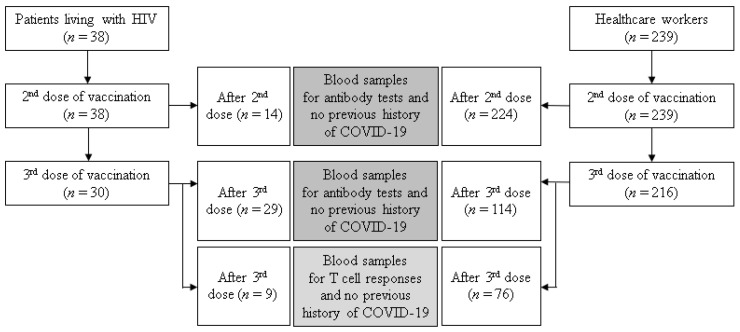
Schematic flow chart of the study.

This was a prospective observational study performed at Chung-Ang University Hospital, Seoul, South Korea. We enrolled PLWH who were on regular follow-up at outpatient clinics and had received a booster dose of COVID-19 vaccine from January 2022 to May 2022. A schematic flowchart of this study is shown in Figure 1. PLWH were defined as patients diagnosed with confirmed HIV infection. PLWH were vaccinated with mRNA (BNT162b2 and mRNA1273) or viral vector (ChAdOx1 nCoV-19 and Ad26.COV2.S) vaccines according to the government’s approved schedules. Blood samples from PLWH were collected after the second or third vaccine dose according to the time of enrollment, if feasible. We collected clinical information including demographic characteristics, time of HIV diagnosis and ART, white blood cell counts, differential counts of lymphocytes, CD4 T cell counts, HIV viral loads, and underlying diseases in PLWH based on electronic medical records. We also included HCWs without HIV who were recruited voluntarily for a study of longitudinal immune responses against SARS-CoV-2 after vaccination from February 2021 to May 2022 in our hospital. All enrolled HCWs have no history of malignancy or immunosuppressive treatment such as chemotherapy, and they take annual health check-ups. HCWs were vaccinated with BNT162b2, mRNA-1273, or ChAdOx1 nCoV-19 according to the approved strategies. Blood samples from HCWs were collected after the second or third vaccination. For subgroup analysis, HCWs were also randomly selected after matching age and sex with PLWH. We described breakthrough cases, which were defined as confirmed COVID-19 after a second or third vaccination. Informed consent was obtained from all participants. The study was conducted in accordance with the principles of the Declaration of Helsinki and approved by the institutional review board of Chung-Ang University Hospital (2111-056-485).

### 2.2. Assessment of SARS-CoV-2 Spike-Specific Immunoglobulin G (IgG)

We performed the Euroimmun anti-SARS-CoV-2 enzyme-linked immunosorbent assay (ELISA) (Euroimmun, Lübeck, Germany) to detect SARS-CoV-2 spike-specific IgG. The results were evaluated by measuring the optical density (OD) at 450 nm, with responses expressed as arbitrary units per milliliter (AU/mL), as previously described in a recent study [15,16]. The result was determined as positive if it was more than 1.1 AU/mL according to the manufacturer’s instructions.

### 2.3. Assessment of Neutralizing Antibody Responses against SARS-CoV-2

We used the GenScript SARS-CoV-2 surrogate virus neutralization test (SVNT) kit (Genscript Biotech Corporation, Piscataway, NJ, USA) to specifically detect neutralizing responses against wild type SARS-CoV-2 as previously described [17]. This test rapidly detects neutralizing antibodies from the participant’s blood sample based on an ELISA by mimicking the interaction between the RBD of the virus and human angiotensin-converting enzyme 2 receptors of the host cell. The percentage of neutralization can be calculated as (1—OD of sample/OD of negative control) × 100, and the recommended positive threshold was 30%. The test was modified to detect neutralizing responses against the RBD of the omicron variant by replacing the horseradish peroxidase-conjugated recombinant RBD fragment according to the manufacturer’s instructions.

### 2.4. Assessment of SARS-CoV-2-Specific T Cell Responses

We also evaluated SARS-CoV-2–specific T cell responses by measuring IFN-γ production after stimulation with SARS-CoV-2 spike protein using the Euroimmun SARS-CoV-2 Interferon Gamma Release Assay (IGRA) (Euroimmun, Lübeck, Germany) according to the manufacturer’s instructions. A set of three tubes was used for each participant: (1) SARS-CoV-2 IGRA BLANK without interferon-activating substance as the individual background stimulation, (2) SARS-CoV-2 IGRA TUBE containing the S1 domain of the SARS-CoV-2 spike protein, and (3) SARS-CoV-2 IGRA STIM containing a mitogen for unspecific interferon stimulation to evaluate the viability and stimulation capacity of T cells and sufficient number of T cells in the participant’s blood sample. The response was defined as IFN-γ concentration in the TUBE sample minus that in the BLANK sample, in international units per milliliter (IU/mL). IFN-γ responses above 200 mIU/mL were defined as positive.

### 2.5. Statistical Analyses

Categorical variables were compared using the chi-square or Fisher’s exact test, as appropriate, and continuous variables were described as median values with interquartile range (IQR) and compared using the Mann–Whitney *U-*test, as appropriate. We used the Kruskal–Wallis rank sum test to compare neutralizing responses against the omicron variant of SARS-CoV-2 according to the days after the third COVID-19 vaccination. A *p* value < 0.05 indicated statistical significance. All statistical analyses were performed using GraphPad Prism version 5.0 and SPSS Statistics version 26 (IBM Corp., Armonk, NY, USA).

## 3. Results

### 3.1. Study Population

A total of 38 PLWH and 239 HCWs, who received a second dose of COVID-19 vaccine, were enrolled in this study. Of these patients, 30 PLWH and 216 HCWs received a third dose of vaccine. To compare humoral and cellular responses between PLWH and HCWs after the third vaccination, we analyzed the results of the tests in 29 PLWH and 114 HCWs for humoral responses and 9 PLWH and 76 HCWs for cellular responses. The clinical characteristics of vaccinated PLWH and HCWs are shown in Table 1 and Supplementary Table S1. Males were more common in PLWH than in HCWs (96.6% vs. 33.3%, *p* < 0.001), and PLWH were older than HCWs (44 vs. 35 years old, *p* = 0.001). Vaccine regimens were not significantly different between two groups. In PLWH, median CD4 T cell counts were 670.0 cells/μL (interquartile range [IQR] 527.1–830.3) and HIV viral loads were suppressed below 20 copies/mL in most (86.2%) of the patients.

### 3.2. SARS-CoV-2 Spike-Specific IgG and Neutralizing Antibody Responses against SARS-CoV-2

**Figure 2:**
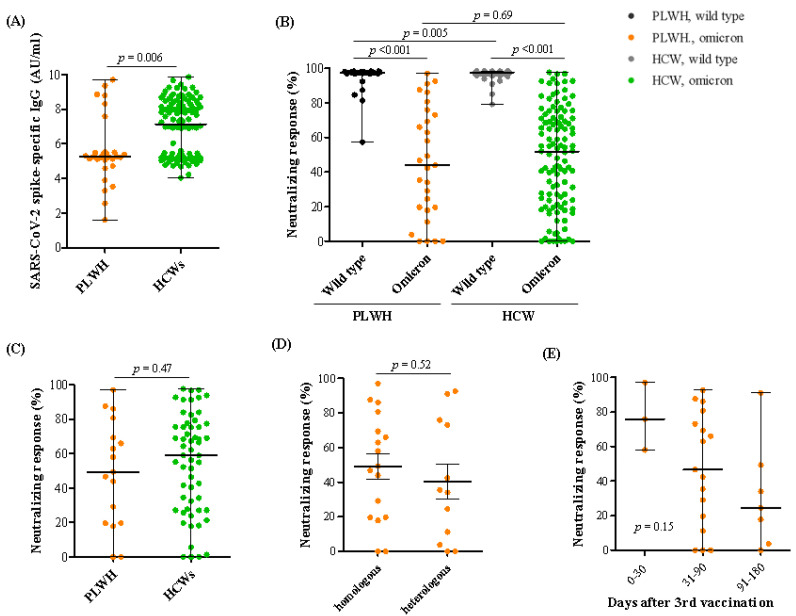
SARS-CoV-2 spike-specific immunoglobulin G (IgG) and neutralizing responses against the wild type and omicron variants of SARS-CoV-2 at median 63 days after a third COVID-19 vaccination in people living with HIV (PLWH) and healthcare workers (HCWs). (**A**) SARS-CoV-2 spike-specific IgG, (**B**) neutralizing responses against wild type and omicron variant, (**C**) neutralizing responses against the omicron variant after a third homologous mRNA vaccination, (**D**) neutralizing responses against the omicron variant in homologous and heterologous vaccinated PLWH, and (**E**) neutralizing responses against the omicron variant according to days after the third vaccination in PLWH.

**Figure 3:**
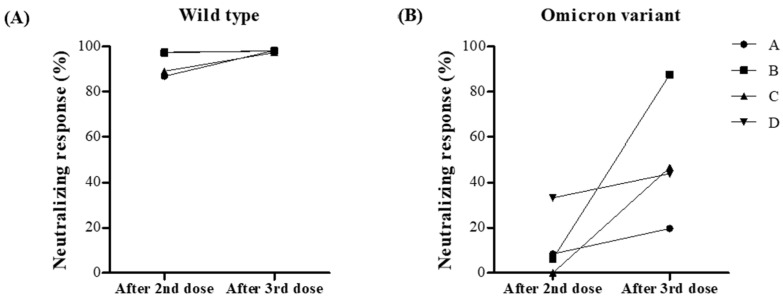
Neutralizing responses against the wild type and omicron variants of SARS-CoV-2 according to number of COVID-19 vaccinations (at median 99 and 57 days after a second and third COVID-19 vaccination, respectively) in four people living with HIV (PLWH). (**A**) Changes of neutralizing responses against the wild type SARS-CoV-2 after the second and third vaccination, (**B**) Changes of neutralizing responses against omicron variant of SARS-CoV-2 after the second and third vaccination.

After the third dose of the COVID-19 vaccine, blood samples were obtained at a median of 63 days in PLWH and HCWs (*p* = 0.36). SARS-CoV-2 spike-specific IgG in PLWH was significantly lower than in HCWs (5.25 vs. 7.14 AU/mL, *p* = 0.006, Figure 2A). In Figure 2B, neutralizing responses against the omicron variant of SARS-CoV-2 were significantly lower than against wild type SARS-CoV-2 in PLWH (97.46% vs. 43.94%, *p* < 0.001) and HCWs (97.74% vs. 51.77% AU/mL, *p* < 0.001). However, neutralizing responses against the omicron variant in PLWH were comparable to those in HCWs (*p* = 0.69). When we compared neutralizing responses against the omicron variant in the subgroup of homologous mRNA vaccinated PLWH, the results showed no significant difference between PLWH and HCWs (*p* = 0.47, Figure 2C). We also analyzed neutralizing responses against the omicron variant between homologous mRNA vaccinated and heterologous vaccinated PLWH, and the results were similar between the two groups (*p* = 0.52, Figure 2D). When analyzing neutralizing responses against the omicron variant according to the duration from the third vaccination to the day of the blood sample tests, the responses had a decreasing tendency related to the duration between vaccination and blood tests, although this was not statistically significant (*p* = 0.15, Figure 2E). We also performed subgroup analysis in 22 PLWH and 22 HCW who were randomly selected by matching age and sex. The results were consistent with those of the total study population (Supplementary Table S2 and Supplementary Figure S1). In PLWH, four patients underwent blood sampling twice after the second and third vaccinations (median 99 and 57 days), and the change in neutralizing responses against the omicron variant seemed to be more remarkable than against the wild type (Figure 3). Detailed information about the time of blood sampling and the results are shown in Supplementary Table S3. We also described SARS-CoV-2 spike-specific IgG and neutralizing responses after the second vaccination, although the time of blood sampling was significantly different between two groups (median 94 days in PLWH vs. 162 days in HCWs, *p* < 0.001) (Supplementary Figure S2).

### 3.3. SARS-CoV-2-Specific T Cell Responses

**Figure 4:**
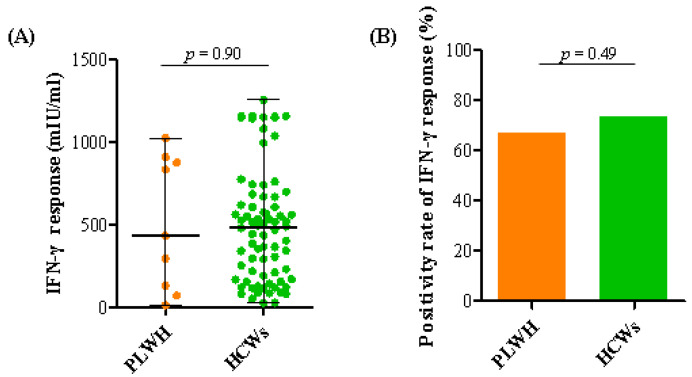
SARS-CoV-2-specific T cell responses at median 65 and 64 days after a third COVID-19 vaccination in people living with HIV (PLWH) and healthcare workers (HCWs). (**A**) Interferon-gamma (IFN-γ) responses and (**B**) positivity rates of IFN-γ releasing assay (IGRA).

We compared IFN-γ levels to evaluate SARS-CoV-2-specific T cell responses in PLWH and HCWs. The tests for IFN-γ production were performed at a median of 65 (IQR 52–152) and 64 (61–118) days (*p* = 0.83) in 9 PLWH and 76 HCWs, respectively. The level of IFN-γ was not significantly different between the two groups (434.84 vs. 484.96 mIU/mL, *p* = 0.90, Figure 4A), and the positivity rate was similar (67.0% vs. 73.7%, *p* = 0.49, Figure 4B).

### 3.4. Vaccine Breakthrough SARS-CoV-2 Infection in the Study Population

Breakthrough SARS-CoV-2 infection cases after the third vaccination occurred in 2 (6.9%) of 29 PLWH and 29 (25.4%) of 114 HCWs. Two PLWH were diagnosed with COVID-19 at 8 and 61 days after the third vaccination. The neutralizing response against the omicron variant was 76.0% in the patient tested before COVID-19 confirmation (at 16 days after the third vaccination).

## 4. Discussion

We compared humoral and cellular immune responses against the omicron variant of SARS-CoV-2 between PLWH with HCWs after the third COVID-19 vaccination. Most PLWH (86.2%) had fully suppressed viral loads and well-controlled CD4 T cell counts (median 670.0 cells/μL). Although SARS-CoV-2 spike-specific IgG and neutralizing responses against wild type SARS-CoV-2 were lower in PLWH than HCWs, neutralizing responses against the currently circulating omicron variant in PLWH were not significantly different from HCWs. We demonstrated that neutralizing responses against the omicron variant were significantly impaired by about 50% compared with wild type SARS-CoV-2 in PLWH and HCWs. In addition, although there was a limitation of small sample size of PLWH, SARS-CoV-2-specific T cell responses determined by measuring the production of IFN-γ were similar between PLWH and HCWs.

When evaluating the effectiveness of a COVID-19 vaccine in PLWH, it is important to consider host and viral factors. It is known that immunogenicity and durability induced by most vaccines in PLWH are impaired compared with healthy controls due to their persistent immune dysregulation and chronic inflammatory status [4,18]. However, humoral and cellular responses induced by COVID-19 vaccination (ChAdOx1 nCoV-19, BNT162b2, and mRNA1273) were reported to be similar in well-controlled PLWH and controls without HIV [5,6,7]. Our study also described comparable neutralizing responses against the omicron variant of SARS-CoV-2, which was the currently dominant variant worldwide, in PLWH compared with HCWs. Although SARS-CoV-2 spike-specific IgG was lower in PLWH than HCWs, vaccine effectiveness for protection against breakthrough SARS-CoV-2 infection correlated with neutralizing responses, not spike-specific IgG [19]. In addition, because the SARS-CoV-2 spike-specific IgG tests, which were known to have correlation with neutralizing responses against the virus, targeted wild type SARS-CoV-2, these results might not definitely predict neutralizing responses against the omicron variant. The median age of both groups was significantly different, however, the possibility of age-related immune-senescence might be considered above 40 years [20]. In addition, the main findings on humoral responses after the third vaccination in subgroup analysis including 22 PLWH and 22 age–sex matched HCWs were consistent with the results in the original groups. Although there might be the possibility of different immune responses according to vaccine regimens (homologous and heterologous), there were no differences according to vaccine regimens between two groups and there were no participants with a homologous adenovirus-vector vaccine regimen. To the best of our knowledge, there is no data on cellular responses after a third vaccination in PLWH. In this study, cellular responses detected by IFN-γ production were similar between PLWH and HCWs. This result was consistent with previous studies showing no difference in cellular immunity after a second vaccination between PLWH and healthy controls [5,21,22]. However, only nine PLWH were included in the analysis of cellular immunity, therefore, it was difficult to be generalizable and draw a firm conclusion.

Since November 2021, when the omicron variant first emerged, this variant has rapidly become the most common circulating strain worldwide. As the COVID-19 pandemic caused by the omicron variant continued, a third vaccination was recommended worldwide due to concerns about waning immunity after two doses of vaccine. However, there were concerns about vaccine-induced immune escape against the omicron variant because this variant has diverse mutations in its spike protein, which is the main target of the currently developed vaccines [23,24]. Real-world data of the general population showed vaccine effectiveness against symptomatic disease caused by the omicron variant after two doses of vaccine (BNT162b2, mRNA1273, and ChAdOx1 nCoV-19) was low (60–70%) at 2 to 4 weeks, and then waned rapidly to less than 20% after 25 weeks; however, it was restored to 60–70% after a third dose of the mRNA vaccine [10]. These results were much lower than those against the delta variant. A recent study showed that humoral responses in PLWH one month after the third vaccination substantially exceeded responses after the second vaccination, although omicron-specific responses were weaker than those against wild type SARS-CoV-2 [14]. Our results were consistent with the results of the previous study. Although our study population was relatively small, we found neutralizing responses against the omicron variant in PLWH were only about half of those against wild type SARS-CoV-2 after a median of 63 days (IQR 36–93) after the third vaccination. In addition, neutralizing responses showed a tendency to decline with time after the third dose, although this was not statistically significant due to the small number of study population.

Based on this study and previous studies, viral factor, not host factor, seems to be key for vaccine effectiveness against the omicron variant in well-controlled PLWH as in HCWs. Even after the third vaccination, neutralizing responses against the omicron variant were insufficient to prevent breakthrough COVID-19, and these might be reduced over time after vaccination in PLWH. In a recent study, the authors demonstrated that neutralizing responses of the newly developed bivalent omicron-containing vaccine (mRNA-1273.214) against the omicron variant were superior to those with mRNA-1273, without significant safety concerns [25]. Therefore, we suggest to provide PLWH as well as the general population with novel vaccines which specifically target emerging variants such as omicron.

This study had some limitations. First, this study was performed in a single center and included a relatively small number of PLWH. Thus, the study results might not be generalizable. Second, most PLWH had fully suppressed HIV viral loads and well-controlled CD4 T cell counts in this study. PLWH with less than 200 cell/mm^3^ of CD4 T cell counts seemed to have impaired vaccine-induced immune responses compared with healthy controls or well-controlled PLWH in a previous study [5]. However, we could not analyze the differences in humoral responses stratified by CD4 T cell counts and viral loads. Third, because confirmed COVID-19 cases were rare in PLWH, the risk factors of breakthrough SARS-CoV-2 infection remain to be determined. Fourth, because the interval from a second vaccination to blood sampling was significantly different between PLWH and HCWs, we could not draw a firm conclusion about humoral responses after the second vaccination. Finally, we could not obtain repeat blood samples after the third vaccine dose in PLWH. Thus, further studies on longitudinal humoral responses after the third vaccination are required to evaluate waning immunity in PLWH.

## 5. Conclusions

In conclusion, humoral and cellular responses against SARS-CoV-2 in PLWH, whose HIV status was well-controlled, were comparable to those of HCWs. However, responses against the omicron variant were much lower than against wild type SARS-CoV-2 in PLWH and HCWs, suggesting that the risk of breakthrough SARS-CoV-2 infection during the pandemic caused by the highly mutated omicron variant could still be high despite three COVID-19 vaccinations. Therefore, considering recent data on safety and immunogenicity of the bivalent omicron-containing vaccine, a booster vaccination with the updated vaccines which targeted new variants are recommended for both PLWH and HCWs.

## Figures and Tables

**Table 1:** Clinical characteristics of third dose vaccinated patients living with HIV and healthcare workers.

	PLWH (n = 29)	Healthcare Workers (n = 114)	*p* Value
Male, n (%)	28 (96.6)	38 (33.3)	<0.001
Age, median (IQR)	44 (34–56)	35 (26–45)	0.001
Vaccine regimen			0.41
Homologous ^a^	17 (58.6)	57 (50.0)	
mRNA	17	57	
Adenovirus-vector	0	0	
Heterologous	12 (41.4)	57 (50.0)	
Ad-Ad-mRNA	8	57	
Ad-mRNA-mRNA	4	0	
HIV status			
Duration after HIV diagnosis, years	11.0 (7.5–13.0)	-	-
Duration after HIV treatment, years	9.0 (6.5–12.0)	-	-
White blood cell, /μL	6200 (5185–7245)	-	-
Lymphocytes, %	36.0 (30.2–42.8)	-	-
CD4 lymphocytes	670.0 (527.1–830.3)	-	-
<20 copies/mL of HIV RNA, n (%)	25 (86.2)	-	-
Underlying diseases or conditions			
Malignancy	4 (13.8)	0	0.001
Chemotherapy	1 (3.4)	0	0.20

HIV, human immunodeficiency virus; PLWH, patient living with HIV; IQR, interquartile range. ^a^ Homologous vaccination with mRNA or adenovirus-vector (Ad) vaccine (e.g., mRNA-mRNA, mRNA-mRNA-mRNA, Ad-Ad, and Ad-Ad-Ad).

## Data Availability

Not applicable.

## Supplementary Materials

The following supporting information can be downloaded at: https://www.mdpi.com/article/10.3390/vaccines10122129/s1, Figure S1: SARS-CoV-2 spike-specific immunoglobulin G (IgG) and neutralizing responses against wild type and omicron variant of SARS-CoV-2 at median 62 days after a third COVID-19 vaccination in 22 people living with HIV (PLWH) and 22 healthcare workers (HCWs) who were randomly selected by matching age and sex with PLWH; Figure S2: SARS-CoV-2 spike-specific immunoglobulin G (IgG) and neutralizing responses against wild type and omicron variant of SARS-CoV-2 after the second dose of COVID-19 vaccination in patients living with HIV (PLWH) and healthcare workers (HCWs); Table S1: Clinical characteristics of vaccinated patients living with HIV and healthcare workers; Table S2. Clinical characteristics of 22 vaccinated patients living with HIV and 22 age-sex matched healthcare workers; Table S3: SARS-CoV-2 spike-specific immunoglobulin G, neutralizing antibody responses and T cell responses against the wild type and omicron variants of SARS-CoV-2 in vaccinated patients living with HIV and healthcare workers.
