# Supplementary material for: Immune Responses against the Omicron Variant of SARS-CoV-2 after a Third Dose of COVID-19 Vaccine in Patients Living with Human Immunodeficiency Virus (PLWH): Comparison with Healthcare Workers
Source: Vaccines (Basel). 2022 Dec 13;10(12):2129. doi: 10.3390/vaccines10122129 (PMC9782380; doi:10.3390/vaccines10122129)

Supplementary Table S1. Clinical characteristics of vaccinated patients living with HIV and healthcare workers

	PLWH (*n* = 38)	Healthcare workers (*n* = 239)	*P* value
Male, n (%)	37 (97.4)	85 (35.6)	<0.001
Age, median (IQR)	41 (32-55)	32 (25-44)	0.001
Vaccination history			
2nd dose	38 (100.0)	239 (100.0)	-
3rd dose	30 (78.9)	216 (90.4)	0.04
Vaccine regimen			0.02
Homologous[a]	24 (63.2)	116 (48.5)	
mRNA	23 (60.5)	114 (47.7)	
Adenovirus-vector	1 (2.6)	2 (0.8)	
Heterologous	13 (34.2)	123 (51.5)	
Ad-Ad-mRNA	9	122	
Ad-mRNA-mRNA	4	1	
Unknown	1 (2.6)	0 (0)	
HIV status			
Duration after HIV diagnosis, years	9.5 (5.0-13.0)	-	
Duration after HIV treatment, years	8.5 (5.0-12.0)	-	
White blood cell, /μl	5,835 (5,035-7,035)	-	
Lymphocytes, %	36.6 (30.9-42.4)	-	
CD4 lymphocytes	586.3 (440.9-804.8)	-	
<20 copies/ml of HIV RNA, n (%)	32/38[b] (84.2)		
Underlying diseases or conditions	7 (18.4)		
Malignancy	4 (10.5)	0	<0.001
Chemotherapy	1 (2.6)	0	0.14

HIV, human immunodeficiency virus; PLWH, patient living with HIV; IQR, interquartile range

[a]Homologous vaccination with mRNA or adenovirus-vector (Ad) vaccine (e.g. mRNA-mRNA, mRNA-mRNA-mRNA, Ad-Ad, and Ad-Ad-Ad)

[b]Of the remaining six patients, four patients had less than 100 copies/ml, one patient had 1,190 copies/ml, and one patient had 1,140,000 copies/ml of HIV RNA

Supplementary Table S2. Clinical characteristics of 22 vaccinated patients living with HIV and 22 age-sex matched healthcare workers

	**PLWH (*n* = 22)**	**Healthcare workers (*n* = 22)**	***P* value**
Male, n (%)	21 (95.5)	21 (95.5)	>0.99
Age, median (IQR)	41 (31-54)	41 (31-51)	0.95
Vaccine regimen			0.13
Homologous[a]	13 (59.1)	8 (36.4)	
mRNA	13	13	
Adenovirus-vector	0	0	
Heterologous	9 (40.9)	14 (63.6)	
HIV status			
Duration after HIV diagnosis, years	10.0 (6.8-13.0)	-	-
Duration after HIV treatment, years	8.5 (6.0-12.0)	-	-
White blood cell, /µl	6,055 (5,020-7,485)	-	-
Lymphocytes, %	37.2 (30.5-44.7)	-	-
CD4 lymphocytes	655.6 (515.2-906.4)	-	-
<20 copies/ml of HIV RNA, n (%)	18 (81.8)	-	-
Underlying diseases or conditions			
Malignancy	2 (9.1)	0	0.49
Chemotherapy	1 (4.5)	0	>0.99

HIV, human immunodeficiency virus; PLWH, patient living with HIV; IQR, interquartile range

[a]Homologous vaccination with mRNA or adenovirus-vector (Ad) vaccine (e.g. mRNA-mRNA, mRNA-mRNA-mRNA, Ad-Ad, and Ad-Ad-Ad)

Supplementary Table S3. SARS-CoV-2 spike-specific immunoglobulin G, neutralizing antibody responses and T cell responses against wild type and omicron variant of SARS-CoV-2 in vaccinated patients living with HIV and healthcare workers

	**PLWH (*n* = 38)**	**Healthcare workers (*n* = 239)**	***P* value**
**After 2nd dose**	n=14	n=224	
Duration between antibody test and last vaccination, days	94 (72-116)	162 (148-164)	<0.001
EUROIMMUN IgG			
Positive, n(%)	14 (100.0)	218 (97.3)	>0.99
Median value, IQR	6.43 (4.61-7.47)	4.18 (2.54-5.69)	0.001
SVNT, wild type			
Positive, n (%)	14 (100.0)	221 (98.7)	>0.99
Median value, IQR	97.28 (92.74-97.49)	88.36 (68.44-96.15)	0.003
SVNT, omicron variant			
Positive, n (%)	3 (21.4)	45/218 (20.6)	>0.99
Median value, IQR	14.50 (6.37-27.66)	21.15 (13.12-27.83)	0.20
Duration between T cell response test and last vaccination, days	90 (71.8-115.5)	-	
IGRA			
Positive, n (%)	5/6 (83.3)	4/43 (9.3)	<0.001
Median value, IQR	551.76 (226.29-935.63)	52.95 (21.38-91.53)	<0.001
**After 3rd dose**	n=29	n=114	
Duration between antibody test and last vaccination, days	63 (36-96)	63 (61-92)	0.36
EUROIMMUN IgG			
Positive, n (%)	29 (100.0)	126 (100.0)	>0.99
Median value, IQR	5.25 (4.89-5.52)	7.14 (5.18-8.32)	0.006
SVNT, wild type			
Positive, n (%)	29 (100.0)	126 (100.0)	>0.99
Median value, IQR	97.46 (96.92-97.84)	97.74 (97.53-98.25)	0.005
SVNT, omicron variant			
Positive, n (%)	18 (62.1)	78 (68.4)	0.42
Median value, IQR	43.94 (18.75-74.55)	51.77 (23.17-71.20)	0.69
Duration between T cell response	65 (52-152)	64 (61-118)	0.83

test and last vaccination, days			
IGRA			
Positive, n (%)	6/9 (67.0)	56/76 (73.7)	0.20
Median value, IQR	434.84 (102.26-893.43)	484.96 (177.00-669.63)	0.90

HIV, human immunodeficiency virus; PLWH, patient living with HIV; IQR, interquartile range; SVNT, surrogate virus neutralization test; IGRA, interferon-gamma releasing assay

Supplementary Figure S1. SARS-CoV-2 spike-specific immunoglobulin G (IgG) and neutralizing responses against wild type and omicron variant of SARS-CoV-2 at median 62 days after a third COVID-19 vaccination in 22 people living with HIV (PLWH) and 22 healthcare workers (HCWs) who were randomly selected by matching age and sex with PLWH.

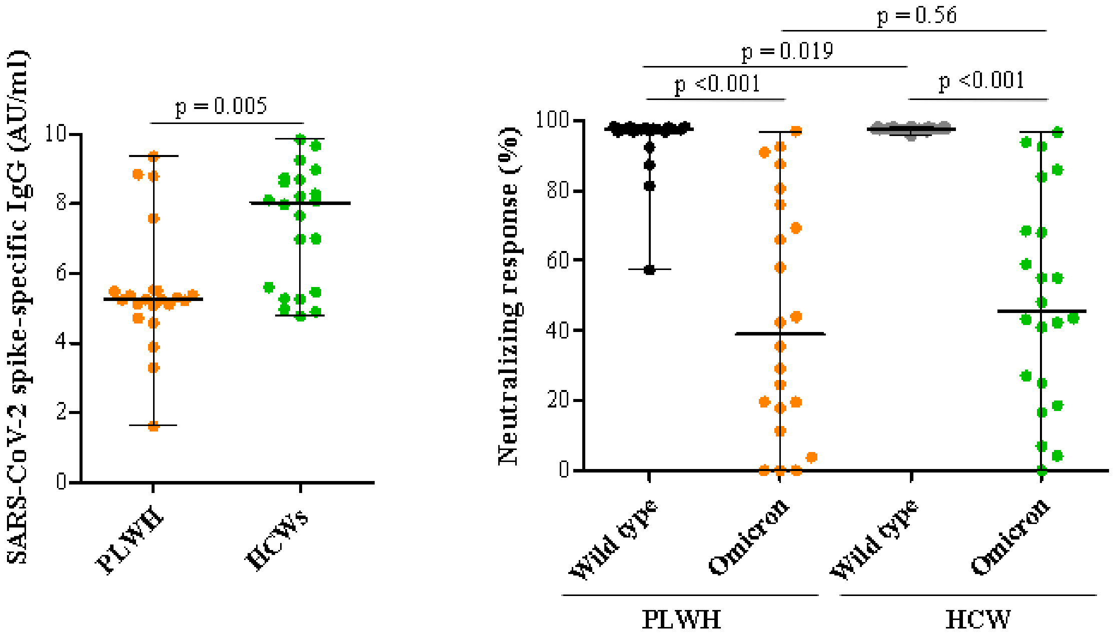

Supplementary Figure S2. SARS-CoV-2 spike-specific immunoglobulin G (IgG) and neutralizing responses against wild type and omicron variant of SARS-CoV-2 after the second dose of COVID-19 vaccination in patients living with HIV (PLWH) and healthcare workers (HCWs)

(A) SARS-CoV-2 spike specific IgG, (B) neutralizing responses against wild type and omicron variant

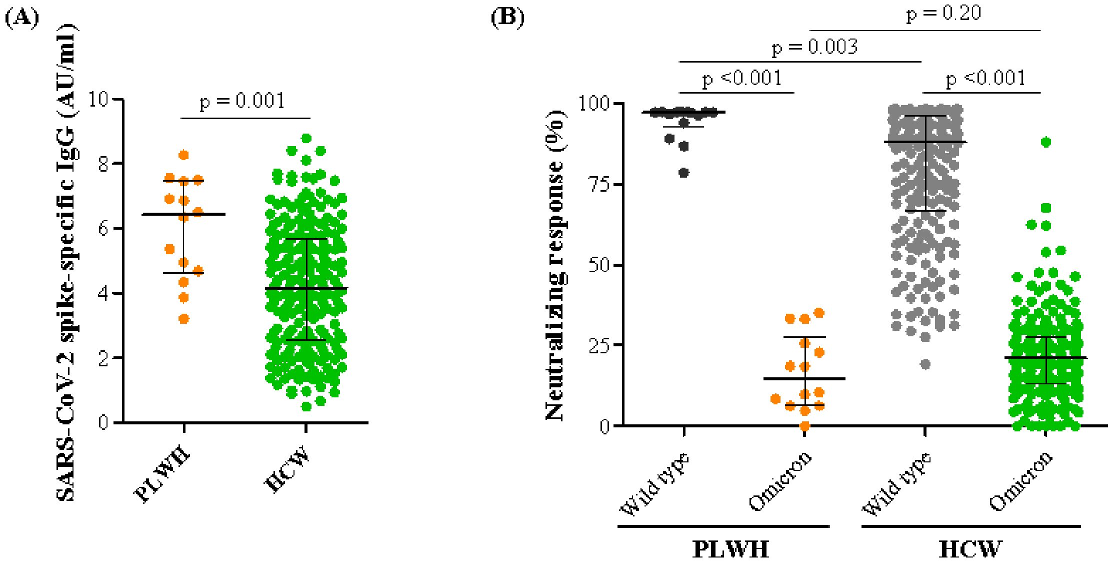

Supplement: Supplementary file 1 [file vaccines-10-02129-s001.zip › vaccines-1992044-supplementary.pdf]
